# Person-environment fit of environmental support provided during medical consultations with older patients

**DOI:** 10.1007/s00391-021-01961-0

**Published:** 2021-09-01

**Authors:** Aoife Poth, Meret Baumgardt, Valentina A. Tesky, Johannes Pantel, Frank Oswald, Julia Haberstroh

**Affiliations:** 1 Interdisciplinary Ageing Research (IAW), Goethe University Frankfurt, Theodor-W.-Adorno-Platz 6, 60323 Frankfurt, Germany; 2 Institute of General Practice in Frankfurt am Main, Goethe University Frankfurt, Theodor-Stern-Kai 7, 60590 Frankfurt, Germany; 3 Psychological Ageing Research (PAR), Faculty II—Education, Architecture, Arts, University of Siegen, Adolf-Reichwein-Str. 2a, 57068 Siegen, Germany

**Keywords:** Supported decision making, Medical consultation, Patient needs, Person-environment fit, Environmental support, Entscheidungsassistenz, Ärztliche Aufklärung, Patientenbedürfnisse, Person-Umwelt-Passung, Unterstützung der Umwelt

## Abstract

As some cognitive functions decline in old age, the ability to decide about important life events such as medical treatment is endangered. Environmental support to improve the comprehension of health-related information is therefore necessary. With a small-scale explorative approach, the present survey study aimed at investigating person-environment fit (PE-fit) of support provided during medical consultations. This fit was calculated by assessing the match between aids provided by five medical practitioners during medical consultations and aids most appreciated by the geriatric patients (*N* = 88). The results showed that the largest discrepancies of used and appreciated aids could be found concerning the opportunity to discuss decisions with relatives, the possibility to take notes, the use of objects, pictures and a keyword list. Female patients indicated a lower PE-fit. These findings highlight discrepancies between the use of specific aids and the wishes of patients and call for thoughtful use of aids during consultations with geriatric patients.

## Abstract (de)

Mit der Abnahme kognitiver Fähigkeiten im Alter ist die Fähigkeit, Entscheidungen über wichtige Lebensereignisse wie etwa medizinische Behandlungen zu fällen, gefährdet. Entscheidungsassistenz ist daher notwendig, um das Verständnis von gesundheitsrelevanten Informationen zu verbessern. Mit einem explorativen Ansatz zielte die vorliegende Befragungsstudie darauf ab, die Person-Umwelt-Passung in der ärztlichen Aufklärung zu untersuchen. Diese Passung wurde berechnet, indem die Übereinstimmung zwischen Hilfestellungen, die von 5 Ärzt:innen während der Aufklärung bereitgestellt wurden, mit den von ihren geriatrischen Patient:innen (*N* = 88) gewünschten Hilfsmitteln erfragt wurde. Die Ergebnisse zeigten, dass die größten Diskrepanzen zwischen verwendeten und gewünschten Hilfsmitteln bei der Möglichkeit, die Entscheidung mit Angehörigen zu besprechen, der Möglichkeit, sich Notizen zu machen und bei der Verwendung von Gegenständen, Bildern und einer Stichwortliste zu finden waren. Patientinnen gaben eine geringere Person-Umwelt-Passung an als Patienten. Diese Befunde verdeutlichen Diskrepanzen zwischen dem Einsatz bestimmter Hilfestellungen und den Wünschen von Patient:innen und legen einen bewussten Einsatz von Hilfsmitteln während ärztlicher Aufklärungen nahe.

## Introduction and background

Article 1 of the universal declaration of human rights states that all human beings are born free and equal in dignity and rights. The freedom of all humans is also embedded in German constitutional law; however, freedom is a broad term and requires further specification.

The global term freedom refers to “freedom of actions” [5]. The central idea behind freedom of actions is that people should be able to shape their own lives and make decisions autonomously [8]; however, to be able to make use of this fundamental right, a person must live in a favorable environment where the resources and options that enable freedom to choose are readily available.

Healthy aging is related to changes in brain and cognition and, whilst differing between individuals, often results in cognitive decline. These functional cognitive changes can result in lower selective information processing speed, diminished cognitive flexibility and in difficulties with so-called dual tasks in which attention must be shared [21, 23]. As working memory and verbal abilities often deteriorate in old age, the right of old people to choose and act autonomously is endangered [2, 3].

Several authors described the particular vulnerability of geriatric patients in the informed consent process [10, 14] due to multimorbidity and susceptibility to coercion, which poses some challenges. Old adults in particular have to make important decisions about health-related issues and must often provide informed consent to medical procedures, for which language comprehension is vital [22]. To maintain the fundamental right to freedom of action as long as possible and ensure self-determined aging, there are approaches to compensate for deficits in the capacity to understand medical information and to decide autonomously by providing adequate environmental support with different aids [9].

Past studies on aids for enhancing the informed consent for a variety of patient groups have yielded mixed results. Meta-analyses on this topic indicate that aids from the categories *extended discussion* and *enhanced consent form* lead to the best effects on the subjects’ understanding of information [6, 15]. Liu et al. [12] investigated the effect of illustrations to improve older adults’ comprehension of health-related information and concluded that visual support was more confusing than helpful. In contrast, Garcia-Retamero and Cokely [7] demonstrated the effectiveness of appropriately designed visual aids. A review by Choi [4] also concluded that the use of pictographs can improve the discharge education of old adults in acute healthcare settings. Despite some contradictory reports, the majority of studies stressed the positive effect of visual aids. Existing research also suggests that older adults’ understanding of health-related information provided during medical consultations may benefit from trusting relationships, a clean and tidy environment, the use of objects, and written information [26].

However, previous studies did not assess the match between the aids patients would generally appreciate and the aids they were provided with. The present study tried to fill this gap by assessing person-environment fit (PE-fit) of 21 different aids from the 4 categories personal aids, room aids, written aids, and object aids, as recommended by Wied et al. [26]. With respect to PE-fit, positive impact on outcomes of autonomy, well-being and health have been repeatedly reported for old adults (e.g. [17, 18]). In order to determine whether environmental support matches patients’ self-reported needs, the aids that were most often employed were compared to those that are most appreciated by the patients.

Within the framework of this exploratory approach, we further examined the PE-fit in relation to psychiatric or neurological pre-existing conditions, and demographic variables such as gender and educational level as predictors of health literacy. Health literacy is considered as “the individual’s capacity to obtain, process, and understand basic health information and services needed to make appropriate health decisions” [20]. It is well-known that gender and low education can affect health literacy and can therefore also be expected to affect medical decision-making [11, 25]. Men have been reported to show lower health literacy than women (e.g. [11, 25]). Furthermore, it has been reported that people with low education levels have lower health literacy than people with high education levels [2, 11, 25].

In summary, the present study aimed at assessing PE-fit in terms of aids used and appreciated during medical consultation and to examine its relationship with demographic variables.

## Study design and investigation methods

### Sample

Recruitment took place in spring and summer 2018 through a network of practices collaborating in research of the Institute of General Practice of Goethe University Frankfurt. An invitation to participate in the study was sent out as part of the regular email newsletter. We included general practitioners and dentists because they are the most frequently consulted practitioners. A total of five practices got back to us and indicated a possible number of their geriatric patients they would approach for the study. Afterwards, 30–50 questionnaires including patient information, consent form and stamped return envelopes were sent by mail to the practices. To ensure comparability of our study with research on supported decision-making in dementia, we only included participants older than 65 years as this is the age from which the most common form of dementia, Alzheimer’s dementia, can be diagnosed according to the ICD-10. Further inclusion criteria were that participants were classified as geriatric patients (defined as typical geriatric multimorbidity or older than 80 years, frailty [24]) and had recently undergone medical consultation with one of the participating practitioners. Participants were excluded from the analysis if they had a diagnosis of dementia and a native language other than German. Of the five practitioners, one was a dentist and four were general practitioners. Two practitioners were women. The age of the practitioners was not assessed in order to ensure anonymity.

Overall, 88 patients completed the pen and paper survey and were included in the analysis. The patients were aged between 65 and 92 years with an average of 74.5 years (*SD* = 6.58), 33% were male, and 55% female, while 12% did not specify their gender. Of the 88 patients, 48 said they had undergone vocational training, 18 had an academic degree, and 22 had a middle school or high school educational level.

### Ethics

All procedures performed in the study were in accordance with the 1964 Helsinki declaration and its later amendments or comparable ethical standards. The study was reviewed by the institutional ethics committee of the University Hospital Frankfurt, which obtained ethical approval in March 2018 (approval number 488/17).

### Material and procedure

The participants were asked to answer the survey after they had undergone a medical consultation in the practice. Consultations mostly referred to diagnostic measures, invasive interventions, medical measures, therapeutic treatments or complex interventions, such as chemotherapy and could therefore take different lengths of time. The questionnaire consisted of 72 items and took about 20 min to fill in. After providing details about themselves, their practitioner and the treatment discussed, participants were asked about the medical consultation. Out of a selection of 21 aids (10 personal aids, 5 room aids, 2 written aids and 4 object aids) that were based on previous studies [11] they were first asked to tick if the aid was used (“yes”) or not (“no”). Afterwards, participants were asked if they generally appreciated the aid (“yes”) or not (“no”).

### Data scoring and statistical analysis

Our study took a small-scale explorative approach to investigating patients’ appreciation of specific aids. The PE-fit scores were determined according to the suggestions of Oswald et al. [16]. Every match between the aid used during the consultation and general appreciation of that aid by the participant was coded with “1”, while every mismatch received a “0”. Missing values were excluded. To assess PE-fit for each aid, the percentage of matches was assessed. To detect differences in PE-fit between patients based on age, gender, pre-existing conditions, and educational level, we calculated Pearson correlations, *t*-tests and analyses of variance (ANOVAs). Dependent variables were overall PE-fit as well as the percentage to which aids were not used but would have been appreciated by a patient.

## Results

Descriptive results are presented per aid category (personal, room, written information, object). Mean overall PE-fit was 84.23% (*SD* = 10.57%), with a minimum of 55.56% and a maximum of 100%. The mean percentage of aids not used but appreciated was 9.69% (*SD* = 9.59%) with a minimum of 0% and a maximum of 38.10%.

Table 1 shows the results for personal aids. The highest discrepancy can be seen between the percentage of patients that were able to discuss the decision with a relative (17%) and the percentage that would generally like to discuss decisions with relatives (76.2%).

**Table 1:** Percentages showing average frequencies of use, appreciation and PE-fit of personal aids

Type of personal aid	Used (%)	Generally appreciated (%)	Used but not appreciated (%)	Not used but appreciated (%)	Overall PE-fit (% matches)
Familiar medical practitioner	95.4	81.0	16.9	2.4	80.7
Personal introduction of medical practitioner	79.2	90.2	4.2	12.5	83.3
Personal greeting by medical practitioner	97.7	96.4	3.6	1.2	95.2
Conversation while seated	97.7	91.8	0	6.0	94.0
Medical practitioner maintains eye contact with patient	98.9	100	0	1.2	98.8
Medical practitioner takes their time	97.7	100	0	2.4	97.6
Important information is repeated	77.5	87.8	3.9	15.6	80.5
Information is divided into short sections	83.8	87.8	7.8	13.0	79.2
No technical terms	91.6	91.8	3.7	4.9	91.4
Opportunity to discuss decisions with relatives	17	76.2	4.3	59.4	36.2

Table 2 shows the results for room aids, which were appreciated by a large percentage of participants, with the exception of consultation at home, which was only appreciated by 1.2%.

**Table 2:** Percentages showing frequencies of reported use, appreciation and PE-fit of room aids

Type of room aid	Used (%)	Generally appreciated (%)	Used but not appreciated (%)	Not used but appreciated (%)	Overall PE-fit (% matches)
Consultation at home	1.1	1.2	1.2	1.2	97.6
Consultation in a quiet room	100	96.5	3.5	0	96.5
Consultation in a bright room	100	84.3	15.7	0	84.3
Consultation in a tidy room	100	89.2	10.8	0	89.2
Consultation without disturbance	97.7	95.3	3.5	1.2	95.3

Table 3 compares the use and appreciation of written aids. Written aids in general were appreciated by a moderate percentage of participants. Both the possibility to take notes and the doctor’s suggestion to take notes were not applied as frequently as would have been appreciated by many patients.

**Table 3:** Percentages showing frequencies of reported use, appreciation and PE-fit of written aids

Type of written aid	Used (%)	Generally appreciated (%)	Used but not appreciated (%)	Not used but appreciated (%)	Overall PE-fit (% matches)
Possibility to take notes	48.1	45.1	18.2	15.6	66.2
Doctor suggested taking notes	4.7	23.5	0	20.0	80.0

Table 4 shows the results for object aids. All of them were relatively rarely used, although many patients would have appreciated them. There appeared to be low demand and a low discrepancy only with respect to videos.

**Table 4:** Percentages showing frequencies of reported use, appreciation and PE-fit of object aids

Type of object aid	Used (%)	Generally appreciated (%)	Used but not appreciated (%)	Not used but appreciated (%)	Overall PE-fit (% matches)
Keyword list	3.6	24.7	0	20.3	79.7
Pictures	14.3	37.0	1.3	26.3	72.5
Objects	20.2	46.3	5.0	32.5	62.5
Videos	0	4.9	0	5	93.8

In order to explore associations between PE-fit and characteristics of the patients, we conducted further analyses. Although the distributions of overall PE-fit and the percentage of aids not used but appreciated were significantly non-normal, skew and kurtosis in both distributions did not exceed 2.0, which according to Miles and Shevlin [13] means the data can nevertheless be assumed to be close to normality.

There was no significant association found between overall PE-fit and age, *r* = 0.165, *p* = 0.125, or between the percentage of aids not used but appreciated and age, *r* = −0.097, *p* = 0.366. As for gender differences in overall PE-fit, homogeneity of variance was given, and while the difference between men (*M* = 87.06%) and women (*M* = 82.53) was only close to being significant, *t*(86) = 1.98, *p* = 0.050, a small to moderate effect size of gender, Cohen’s *d* = 0.437, was found. Similarly, there was a small gender difference on the percentage of aids not used but appreciated. Women (*M* = 11.26%) stated significantly more often than men (*M* = 7.07%) that they would have appreciated an aid that was not used, *t*(86) = −2.02, *p* = 0.047. Here, too, a small to moderate effect size of gender was found, Cohen’s *d* = −0.444.

Tests on the association between the patients’ educational level, which is related to their health literacy, and their reported overall PE-fit revealed that there were no significant differences between patients with different educational levels, *F*(2, 85) = 0.658, *p* =0.520. Similarly, no such differences were found concerning the percentage of aids not used but appreciated, *F*(2, 85) = 1.382, *p* = 0.257.

Finally, no difference in overall PE-fit could be found between patients who had a pre-existing psychiatric or neurological condition (*M* = 84.68%) and those who did not (*M* = 84.06%), *t*(86) = −0.247, *p* = 0.806. The same was true concerning the percentage of aids not used but appreciated, whereby patients who had a pre-existing psychiatric or neurological condition (*M* = 9.71%) and those who did not (*M* = 9.67%) did not differ significantly, *t*(86) = −0.017, *p* = 0.986.

## Discussion

The present study is one of the first to investigate the PE-fit of environmental support during medical consultations. For this purpose, geriatric patients were questioned on the use and their appreciation of 21 aids. The most striking result is the large discrepancy between patients who were provided with the opportunity to discuss medical decision with relatives and those who would have wished to do so. Furthermore, it was observed that many patients would prefer increased use of objects, pictures and a keyword list during the consultation, and that many would have liked the suggestion and the possibility to take notes.

Personal aids appeared to be important to almost all participants. Social interaction might help patients feel less insecure, as medical consultations can then be seen as an institutionalized communicative practice in which the doctor tries to minimize a patient’s uncertainty by delivering their advice in a reassuring way [19].

Previous studies have shown that consultation room design has an impact on the interaction between doctor and patient [1, 27]; however, these studies examined room designs with enhanced technology. As most participants in our study appreciated more basic room characteristics such as a tidy, calm and bright environment, it can be hypothesized that even these may have an influence on the decision-making process. It would therefore be interesting to examine the impact of consultation room design on outcome measures, such as participants’ understanding of information and satisfaction with health-related decisions.

Aids that are only appreciated by a few patients may not be generally suitable for use during medical consultations. For example, the use of videos may be too time-consuming and therefore more tiring than helpful for patients. Consultations at home would only have been appreciated by one participant. This suggests that geriatric patients find consultations at home too intrusive.

Explorative inferential analyses showed that there were no associations between age, educational level or the presence of a pre-existing psychiatric or neurological condition on PE-fit; however, analyses revealed a small to moderate effect of gender on PE-fit. Women on average indicated a lower overall PE-fit and stated more often than men that they would have appreciated an aid that was not used. This implies that the aids used by the practitioners were less in line with what female patients would generally appreciate than was the case with male patients. It could be speculated that the consultation is more adapted to the needs of male patients than to those of female patients. The discussion about the observed gender differences could also be extended to the current debate on discrimination against women in the healthcare system. Either way, these gender effects offer an interesting starting point for investigating patient’s needs and thoughts about aids and their use in more depth.

The present study has some limitations. Because the sample was so small, it cannot be seen as representative. Only five practitioners responded to our invitation to the study, which is why we must assume that they form a very selective group who are specifically interested in the topic. The participants, being mobile enough to visit the practice and competent enough to fill in the questionnaire, also form a selective group. Furthermore, we did not control for our inclusion criterion geriatric patient but delegated this judgement to the recruiting practitioners. We acknowledge that the selectiveness of both practitioners and patients as well as the patients’ individual medical history, diagnosis and relationship with the practitioner might have influenced the patients’ responses in the questionnaire; however, the selectivity of the groups, and the discrepancy that nevertheless exists between used and appreciated aids, suggest that in the average population of geriatric patients and practitioners, the need for aids and research on PE-fit may be even higher. Moreover, 76.6% of the patients reported having a psychological, psychiatric, neurological or medical condition. It is quite possible that these conditions might be related to preferences for certain aids in different ways, for example less appreciation for written aids or visual aids when vision is impaired. Further research should consider these links.

Furthermore, it should also be mentioned that for the calculation of the PE-fit, the number of aids used by a practitioner did not matter. Thus, a practitioner who used very few aids could score just as high on overall PE-fit as a practitioner who used a lot of aids, but whose aids did not match the ones appreciated by their patient; however, the number of aids used might indeed be relevant for the general perception of support. A more detailed analysis of practitioners’ habits of using aids might therefore also be useful in a possible replication of the study.

As we did not specify whether “not appreciated” meant that patients did not see the aid as necessary or that they actually disliked it, our study lacks detailed information on patients’ associated feelings and thoughts. Future surveys asking patients for their perspectives and reasons for their preferences may be useful. Moreover, effectiveness of aids, perhaps operationalized as patients’ understanding of treatment information or satisfaction with the decision, could not be determined.

Our findings enable some recommendations for future research and practice. Family members should be integrated more often into medical decision-making procedures involving old patients. As many patients appreciate personal and room aids, medical practitioners should continue to personally introduce themselves, offer a personal greeting, lead the conversation while seated, maintain eye contact, take their time, divide information into sections and repeat important information, avoid technical terms, and make sure the consultation takes place in a tidy, clean and bright room. Practitioners should offer patients the opportunity to discuss decisions with relatives, suggest that their patients take notes, and consider the use of object aids, such as a keyword list with bullet points on the most important information, pictures, and objects such as the package of a drug when pharmacological treatment is discussed.

Although PE-fit was mostly moderate to high, the present study unveiled some discrepancies between aids used and those appreciated by patients. This implies that while the use of aids is important, there appears to be no universal solution for all patients. A possible approach could be to ask patients about their preferences and needs before implementing aids. In this case, it must be evaluated whether this supports patients or whether the offer could overwhelm them.

Possible replication studies should include a higher number of practitioners and a bigger overall sample. The type of practitioner and the type of treatment discussed should be included in the analysis in order to detect specificities of PE-fit for different disciplines and diagnoses. Future research should test the effect of aids on a variety of health-related outcome measures.

In conclusion, the present study showed that there are discrepancies between the aids that general practitioners and dentists use and those that their geriatric patients would wish for. We hope the present study can serve as a basis for the generation and testing of further hypotheses concerning the use of certain aids during consultations in medical practice.

- Practitioners should keep up to date with the many ways to support old patients during consultations and use aids in an evidence-based way.
- Practitioners should stay aware that personal aids such as a personal introduction, the repetition of important information, or the division on information into short sections, are important to most patients and should be routinely implemented.
- A majority of patients would appreciate the opportunity to discuss medical decisions with relatives. Practitioners should try and enable them to do so.

## References

[1] Ajiboye F, Dong F, Moore J, Kallail KJ, Baughman A (2015). Effects of revised consultation room design on patient–physician communication. Health Environ Res Des J.

[2] Benson J, Foreman WB (2002). Comprehension of written health care information in an affluent geriatric retirement community: use of the test of functional health literacy. Gerontology.

[3] Brown SC, Park DC (2003). Theoretical models of cognitive aging and implications for translational research in medicine. Gerontologist.

[4] Choi J (2011). Literature review: using pictographs in discharge instructions for older adults with low-literacy skills. J Clin Nurs.

[5] Epping V (2012). Kapitel 11: Allgemeine Handlungsfreiheit (Art. 2 Abs. 1 GG). Grundrechte.

[6] Flory J, Emanuel E (2004). Interventions to improve research participants’ understanding in informed consent for research: a systematic review. JAMA.

[7] Garcia-Retamero R, Cokely ET (2013). Communicating health risks with visual aids. Curr Dir Psychol Sci.

[8] Gumbis J, Bacianskaite V, Randakeviciute J (2008). Do human rights guarantee autonomy?. Cuadernos Const Cátedra Fadrique Furió Ceriol.

[9] Haberstroh J, Oswald F (2014). Unterstützung von Autonomie bei medizinischen Entscheidungen von Menschen mit Demenz durch bessere Person-Umwelt-Passung? [Enhancing autonomy in medical decision making among people with dementia by improving person-environment fit. Informationsd Altersfr.

[10] Ivashkov Y, Van Norman GA (2009). Informed consent and the ethical management of the older patient. Anesthesiol Clin.

[11] Kobayashi LC, Wardle J, Wolf MS, von Wagner C (2015). Cognitive function and health literacy decline in a cohort of aging English adults. J Gen Intern Med.

[12] Liu CJ, Kemper S, McDowd J (2009). The use of illustration to improve older adults’ comprehension of health-related information: Is it helpful?. Patient Educ Couns.

[13] Miles J, Shevlin M (2001). Applying regression and correlation: a guide for students and researchers.

[14] Mukherjee A, Livinski AA, Millum J, Chamut S, Boroumand S, Iafolla TJ, Adesanya MR, Dye BA (2017). Informed consent in dental care and research for the older adult population. J Am Dent Assoc.

[15] Nishimura A, Carey J, Erwin PJ, Tilburt JC, Murad MH, McCormick JB (2013). Improving understanding in the research informed consent process: a systematic review of 54 interventions tested in randomized control trials. BMC Med Ethics.

[16] Oswald F, Hieber A, Wahl HW, Mollenkopf H (2005). Ageing and person-environment fit in different urban neighbourhoods. Eur J Ageing.

[17] Oswald F, Konopik N (2015). Bedeutung von außerhäuslichen Aktivitäten, Nachbarschaft und Stadtteilidentifikation für das Wohlbefinden im Alter. Z Gerontol Geriatr.

[18] Oswald F, Wahl HW (2019). Physical contexts and behavioral aging.

[19] Pilgram R (2009). Argumentation in doctor-patient interaction: medical consultation as a pragma-dialectical communicative activity type. Stud Commun Sci.

[20] Ratzan SC, Parker RM, Selden CR, Zorn M, Ratzan SC, Parker RM (2000). Introduction. National library of medicine current bibliographies in medicine: health literacy.

[21] Salthouse TA (2009). When does age-related cognitive decline begin?. Neurobiol Aging.

[22] Schatz T, Haberstroh J, Bindel K, Oswald F, Pantel J, Paulitsch M, Konopik N, Knopf M (2017). Improving comprehension in written medical informed consent procedures. GeroPsych.

[23] Schipper M, Scherenberg V (2017). Kognitive Veränderungen im Alter: Förderung kognitiver Kontrolle als präventiver Ansatz?. Public Health Forum.

[24] Sieber CC (2007). The elderly patient—who is that?. Internist.

[25] von Wagner C, Knight K, Steptoe A, Wardle J (2007). Functional health literacy and health-promoting behavior in a national sample of British adults. J Epidemiol Community Health.

[26] Wied TS, Knebel M, Tesky VA, Haberstroh J (2019). The human right to make one’s own choices–implications for supported decision-making in persons with dementia. Eur Psychol.

[27] Zamani Z, Harper EC (2019). Exploring the effects of clinical exam room design on communication, technology interaction, and satisfaction. Health Environ Res Des J.

